# Interplays between *cis*- and *trans*-Acting Factors for Alternative Splicing in Response to Environmental Changes during Biological Invasions of Ascidians

**DOI:** 10.3390/ijms241914921

**Published:** 2023-10-05

**Authors:** Xuena Huang, Hanxi Li, Aibin Zhan

**Affiliations:** 1 Research Center for Eco-Environmental Sciences, Chinese Academy of Sciences, 18 Shuangqing Road, Haidian District, Beijing 100085, China; xnhuang@rcees.ac.cn (X.H.); lihanxi1021@163.com (H.L.); 2 University of Chinese Academy of Sciences, 19A Yuquan Road, Shijingshan District, Beijing 100049, China

**Keywords:** alternative splicing, biological invasion, genome architecture, SRSFs, stress response

## Abstract

Alternative splicing (AS), a pivotal biological process contributing to phenotypic plasticity, creates a bridge linking genotypes with phenotypes. Despite its importance, the AS mechanisms underlying environmental response and adaptation have not been well studied, and more importantly, the *cis*- and *trans*-acting factors influencing AS variation remain unclear. Using the model invasive congeneric ascidians, *Ciona robusta*, and *Ciona savignyi*, we compared their AS responses to environmental changes and explored the potential determinants. Our findings unveiled swift and dynamic AS changes in response to environmental challenges, and differentially alternative spliced genes (DASGs) were functionally enriched in transmembrane transport processes. Interestingly, both the prevalence and level of AS in *C. robusta* were lower than those observed in *C. savignyi*. Furthermore, these two indices were higher under temperature stresses compared to salinity stresses in *C. savignyi*. All the observed patterns underscore the species-specific and environmental context-dependent AS responses to environmental challenges. The dissimilarities in genomic structure and exon/intron size distributions between these two species likely contributed to the observed AS variation. Moreover, we identified a total of 11 and 9 serine/arginine-rich splicing factors (SRSFs) with conserved domains and gene structures in the genomes of *C. robusta* and *C. savignyi*, respectively. Intriguingly, our analysis revealed that all detected SRSFs did not exhibit prevalent AS regulations. Instead, we observed AS control over a set of genes related to splicing factors and spliceosome components. Altogether, our results elucidate species-specific and environmental challenge-dependent AS response patterns in closely related invasive ascidians. The identified splicing factors and spliceosome components under AS control offer promising candidates for further investigations into AS-mediated rapid responses to environmental challenges complementary to SRSFs.

## 1. Introduction

Understanding how organisms functionally respond to environmental changes is fundamental for elucidating adaptation mechanisms and further predicting their future performance in ecological processes, such as global climate change and biological invasions. Alternative splicing (AS), a crucial biological process bridging genotypes and phenotypes, has been widely proposed as a mechanism that facilitates stress response and adaptive evolution by expanding transcriptomic and proteomic diversity [1,2]. Notably, recent studies have revealed that AS plays independent or complementary roles to gene expression regulation mechanisms [3,4]. Moreover, the prominent species-specific AS patterns versus tissue-specific gene expression profiles in recently diverged lineages highlight that AS evolves more rapidly than gene expression variation over short timescales [2,5,6]. These recent findings underscore the importance of integrating AS with gene expression to establish a comprehensive framework for investigating the mechanisms of phenotypic plasticity and subsequent rapid adaptive evolution. To construct such a framework, it is imperative to profile species-specific AS patterns in response to environmental changes in closely related species and further identify the common and species-specific regulators responsible for AS variation.

*Ciona robusta* and *C. savignyi*, two congeneric tunicates with similar morphology, have long served as model organisms in multi-disciplines such as evolutionary and developmental biology. Due to their significant invasion records globally, they have become model systems for invasion science [7], particularly for studying the mechanisms underlying invasion success [8]. These species typically co-occur in the same habitats but employ distinct ecological strategies to counteract environmental fluctuations and challenges during invasions. For instance, *C. savignyi* invested more energy in growth to enhance the efficiency of food filtering, whereas *C. robusta* allocated more resources to thickening its tunic to reduce predation risks [9]. The cumulative evidence, including modeling analyses and empirical assessments such as successful invasions into the Red Sea, clearly indicates the rapid adaptive capacity of these two *Ciona* ascidians in the face of adverse and harsh environments [10,11]. Previous studies have comprehensively investigated their environmental stress-coping mechanisms at multiple layers, encompassing genetic adaptation [10,12], epigenetic modification [13,14], transcriptional variation [15,16], proteomic response [17,18], and physiological change [17,18]. Remarkably, we observed that, in conjunction with other regulatory mechanisms such as gene expression and alternative polyadenylation, AS independently acted on different gene sets and associated biological functions in response to environmental challenges during invasions [15,16]. These findings have paved the way for further exploration into the functional roles of AS in responding to environmental challenges during biological invasions, particularly regarding whether AS-based responses are conserved or diverse among congeneric species and further the influential factors contributing to the observed AS patterns.

AS is a complex process orchestrated by the intricate spliceosome, which precisely identifies splicing signals on pre-mRNA and catalyzes the removal of introns to form mature mRNA [19]. A crucial step in AS is the recognition of splice sites by splicing factors and subsequent spliceosome assembly [1]. Consequently, AS variation can result from mutations in both *cis*-regulatory elements and *trans*-acting factors [20]. *Cis*-regulatory elements encompass canonical splice sites as well as exonic and intronic splicing enhancers/silencers (ESEs, ESSs, ISEs, and ISSs), which are primarily recognized and bound by RNA-binding proteins (*trans*-acting factors). These *trans*-acting factors include serine/arginine-rich splicing factors (SRSFs) and heterogeneous nuclear ribonucleoproteins (hnRNPs) that activate or repress splicing [21,22]. SRSFs, as major splicing regulators, are part of the serine arginine-rich protein family and are evolutionarily conserved among higher eukaryotes. They typically contain at least one RNA recognition motif (RRM domain) at the N-terminal and an RS domain enriched with Arginine (R) and Serine (S) at the C-terminal [21,23]. During splicing, SRSFs bind to ESEs via the RRM domain and recruit other spliceosome components via protein–protein or protein-RNA interactions mediated by the RS domain [24]. Thus, SRSFs play crucial roles in splicing functions and responses to environmental changes. For instance, SRSFs themselves are the targets of AS regulations, with the poison cassette exon splicing of SRSF6 downregulating its expression and regulating nuclear speckle dispersal to maintain genomic stability during AS responses to hypoxia [25]. It is noteworthy to mention that the number of SRSF family members may increase with more complex AS profiles [21]. Therefore, genome-wide identification and systematic analysis of SRSFs are expected to shed light on AS changes. In addition to the aforementioned *cis*- and *trans*-acting factors, genomic architecture features such as exon–intron structures and exon/intron length can significantly contribute to AS variation [6]. As the number of exons per gene increases, so does the potential for AS, whereas the length of introns within a genome is believed to be positively correlated with both the level and type of AS [26,27]. Altogether, it is hypothesized that *cis*- and *trans*-acting factors jointly contribute to AS changes in response to environmental challenges.

In this study, we utilized invasive *C. robusta* and *C. savignyi* as models to assess the prevalence, types, and response patterns of AS to environmental challenges (salinity and temperature stresses) during biological invasions. Our objectives were to determine if different AS profiles exist between closely related species exposed to varying environmental cues and to investigate the mechanisms underlying the observed AS variation, primarily from the perspectives of genomic structure architecture and SRSFs.

## 2. Results

### 2.1. AS Profiles in Response to Recurrent High Salinity (HS) Stresses in C. robusta

**Figure 1:**
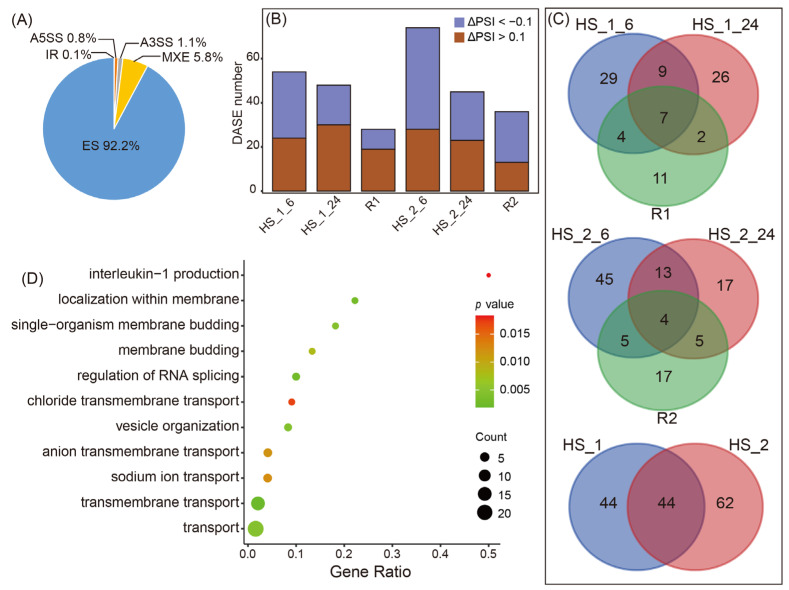
Genome-wide alternative splicing profile of *Ciona robusta* in response to recurrent high salinity challenges. (**A**) Percentages of five main AS types; (**B**) the number of differentially alternatively spliced events (DASEs) in six heat stress (HS)-treated groups compared with the control group, where ΔPSI < −0.1 indicates DASEs in which an exon is skipped, and ΔPSI > 0.1 indicates DASEs in which an exon is obtained under HS challenges; (**C**) comparisons of DASGs at different phases of HS stresses using Venn diagram; (**D**) “Biological process” category of Gene ontology (GO) enrichment analysis of DASGs.

We identified five fundamental types of alternative splicing events (ASEs), namely exon skipping (ES), mutually exclusive exons (MXE), alternative 3′ splice sites (A3SS), alternative 5′ splice sites (A5SS), and retained introns (IR). These ASEs accounted for 92.2%, 5.8%, 1.1%, 0.8%, and 0.01% of all ASEs, respectively (Figure 1A). Given that ES overwhelmingly dominated in terms of abundance, our subsequent analyses focused exclusively on this particular type. Following the initial “HS stress-recovery” challenge, we identified 2755, 2286, and 2429 ES events at 1 h, 6 h post-HS stresses, and 24 h after recovery, respectively. For the subsequent round of the “HS stress-recovery” challenge, we revealed 3272, 2802, and 2911 ES events at the corresponding timepoints. Among these ES events, significant changes in the inclusion level of the target exon (ΔPSI) occurred in only 28 to 74 exons between the treatment and control groups (|ΔPSI| > 0.1 and adjusted *p* value < 0.05; Figure 1B). These specific events were classified as differentially alternatively spliced events (DASEs). Notably, we observed a similar number of DASEs that included an exon (50%, ΔPSI < −0.1) as those involving exon skipping (50%, ΔPSI > 0.1). These DASEs were associated with 150 genes, referred to as differentially alternatively spliced genes (DASGs). Throughout various phases of high salinity stresses (Figure 1C), we detected distinct DASGs, indicating the highly dynamic nature of stress-induced AS regulation. Our GO enrichment analysis of the 150 DASGs showed that there were 20 genes related to the biological processes of transmembrane transport and three genes were linked to RNA splicing regulation (Figure 1D).

### 2.2. Comparisons of AS Profiles and Genomic Features between C. robusta and C. savignyi

**Figure 2:**
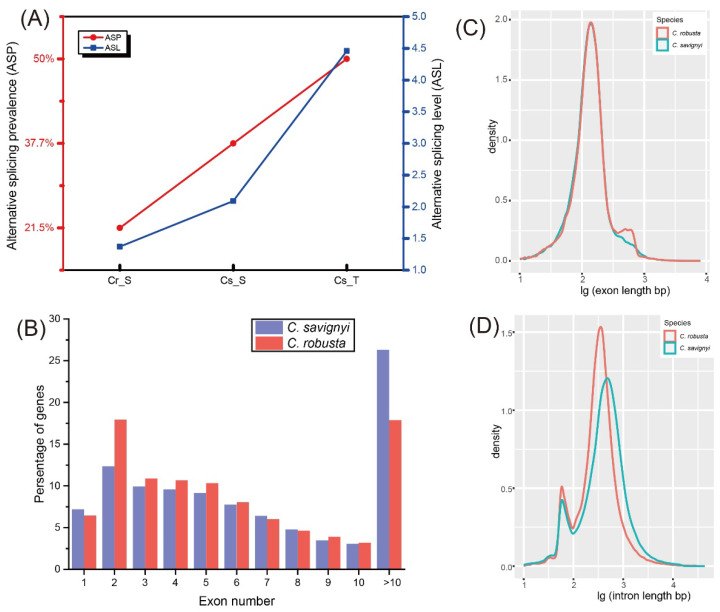
Comparisons of alternative splicing prevalence/level and genomic traits between *Ciona robusta* and *C. savignyi*. (**A**) Alternative splicing prevalence (ASP) and alternative splicing level (ASL) under different challenges. Cr_S: *C. robusta* under recurrent high salinity stresses; Cs_S: *C. savignyi* under salinity stresses; Cs_T: *C. savignyi* under temperature stresses; (**B**) distribution of the number of exons per gene across the whole genome of *C. robusta* and *C. savignyi*, and all genes with more than ten exons are merged; (**C**) distributions of lg (exon length); (**D**) distributions of lg (intron length).

We observed a higher proportion of ES events in *C. robusta* (92.2%) compared to *C. savignyi*, where it accounted for 70.7% under salinity stresses and 51.8% under temperature stresses (Figure 1A and Table S1). To further delve into the AS differences between the two species, we compared alternative splicing prevalence (ASP) and alternative splicing level (ASL) under different environmental stresses (Figure 2A). The results showed that these two AS parameters exhibited variations depending on the species and environmental factors. Specifically, ASP (21.5%) and ASL (1.37) in *C. robusta* under recurrent high salinity (HS) stresses were lower than those observed in *C. savignyi* under both salinity (ASP: 37.7%; ASL: 2.09) and temperature (ASP: 50%; ASL: 4.46) stresses. Notably, temperature stresses induced higher ASP and ASL in *C. savignyi* compared to the salinity stresses (Figure 2A).

To understand the potential genomic features contributing to the differential AS profiles between the two species, we explored several key factors, including the number of exons per gene and the length of exons and introns. Both species primarily exhibited genes with two exons, with *C. savignyi* showing a higher proportion of genes with more than ten exons (Figure 2B). Genes containing a single exon were functionally enriched in various RNA processing functions, such as RNA expression and RNA splicing (Figure S1A,B), whereas genes with over 10 exons were enriched in functions related to signal transduction, stress response, transmembrane transport, among others (Figure S1C,D). Regarding the distribution of exon length, *C. robusta* and *C. savignyi* displayed similar patterns, with a prominent peak at 138 bp and 141 bp, respectively, whereas an additional minor peak appeared at 497 bp in *C. robusta* (Figure 2C). In terms of intron size distribution, both species exhibited a bimodal distribution (Figure 2D), with the major peak being approximately three times more densely populated than the minor one. Notably, while their smaller introns had similar sizes (58 bp for *C. robusta* versus 59 bp for *C. savignyi*), the intron length of the major peak in *C. savignyi* (485 bp) was notably longer than that in *C. robusta* (372 bp) (Figure 2D).

### 2.3. Trans-Splicing Factors under AS Regulation

In *C. robusta* under recurrent high salinity stresses, we identified three DASGs that are functionally associated with the AS process. These genes include *hnRNPLL*, *hnRNPK*, and *CLK2* (Table 1). However, in *C. savignyi* exposed to high salinity stresses, we did not detect any potential *trans*-splicing factors that met the criteria to be classified as DASGs. Additionally, we conducted an analysis in *C. savignyi* under low salinity and both high- and low-temperature stresses. We identified and compiled a list of genes associated with the splicing process that exhibited significantly differential AS profiles under these conditions (Table 1).

### 2.4. Genome-Wide Identification of SRSF Members

In our analysis, a total of nine SRSF genes were identified from *C. savignyi*. These included two *Cs_SRSF1*, one *Cs_SRSF2*, one *Cs_SRSF3*, two *Cs_SRSF6*, two *Cs_SRSF7*, and one *Cs_SRSF12* (Table 2). Interestingly, *C. robusta* possessed two additional members of SRSF6, in addition to the orthologs of the genes listed above. The predicted SRSF proteins exhibited a range in length from 149 to 342 amino acids (AAs). They all contained at least one RNA recognition motif (RRM) domain, in addition to a low complexity region. Notably, the density of SR/RS repeats in the low complexity region exceeded 49% within a sliding window of 30 AAs. Importantly, this characteristic did not show any significant difference between the two species (Table 2).

**Figure 3:**
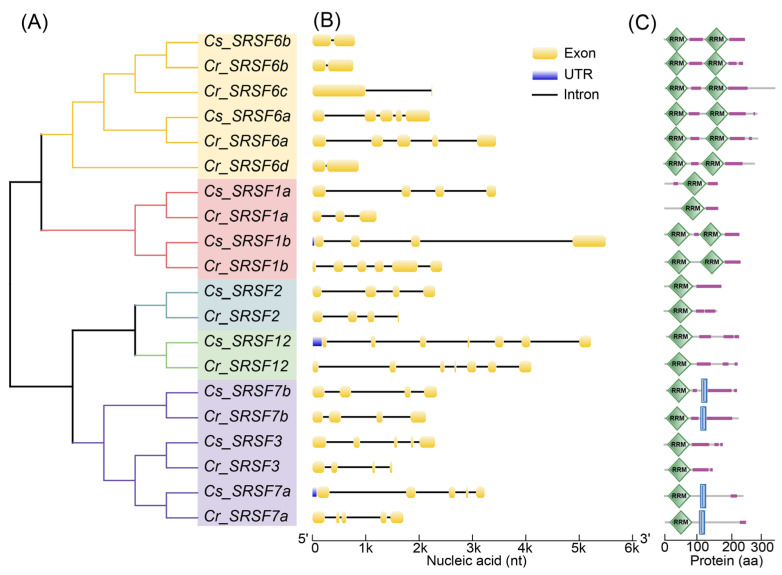
Phylogenetic analysis, exon–intron structure, and conserved domains of serine/arginine-rich splicing factor (SRSF) genes in *C. robusta* and *C. savignyi*. (**A**) Unrooted maximum likelihood (ML) tree. The five shaded clusters indicate the known conserved SRSF subfamilies: yellow for SRSF4/5/6, pink for SRSF1/9, blue for SRSF2/8, green for SRSF10/12, and purple for SRSF3/7. (**B**) exon–intron structures of SRSF genes. (**C**) Protein domains of SRSFs. The green square, purple line, and vertical blue rectangle indicate the RNA recognition motif (RRM) domain, low-complexity region, and ZnF_C2HC domain, respectively.

To elucidate the phylogenetic relationship of SRSF proteins between *C. robusta* and *C. savignyi*, we constructed a maximum likelihood (ML) tree. This analysis revealed five distinct SRSF gene subfamilies (Figure 3A): SRSF1/9, SRSF2/8, SRSF3/7, SRSF4/5/6, and SRSF10/12. Initially, each SRSF gene member in *C. robusta* was clustered with its orthologous counterpart in *C. savignyi*. Subsequently, SRSF1/9 was further clustered with the SRSF4/5/6 subfamily, whereas SRSF2/8 initially grouped with SRSF10/12 before forming a cluster with the SRSF3/7 subfamily. Given that exon–intron organization plays a crucial role in determining the AS profiles of SRSF genes themselves, we assessed the gene structures of SRSFs. We observed that the number of exons ranged from two (SRSF6) to seven (SRSF12). Interestingly, while they generally shared similar exon–intron organization in orthologous counterparts, the length of most introns in *C. savignyi* SRSF genes was longer compared to those in *C. robusta* (Figure 3B). Additionally, we analyzed the domains present in SRSF proteins. All SRSF proteins contained one or two RRM domains, in addition to a low complexity region (SR/RS). Notably, all SRSF6 proteins and one of the SRSF1 proteins possessed two RRMs. Furthermore, SRSF7 proteins were distinguished by an additional ZnF_C2HC domain (Figure 3C). These results indicated that each SRSF subfamily shared a similar protein domain composition and structure, underscoring their highly conserved functions between the two species.

### 2.5. Gene Expression and AS Responses of SRSF to Environmental Changes

**Figure 4:**
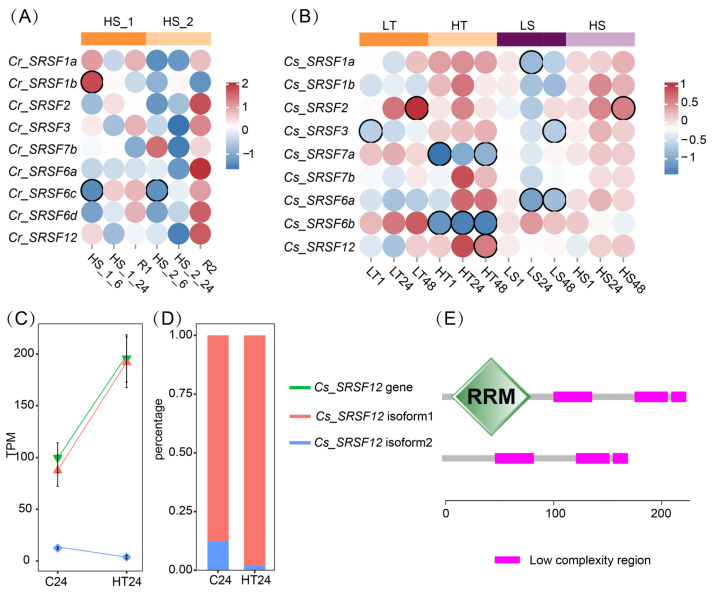
Gene expression and alternative splicing response of serine/arginine-rich splicing factor (SRSF) genes to environmental changes. (**A**) SRSF gene expression changes under recurrent high salinity stresses in *C. robusta*, of which *Cr_SRSF7a* and *Cr_SRSF6b* genes were excluded from differential expression analysis due to their low expression level. The Log_2_ foldchange values between treatment and control groups were used to draw the heatmap, and the color circles with black borders indicate significantly changed genes (adjusted *p* value < 0.05). (**B**) SRSF gene expression changes under low (LT) and high temperature (HT), and low (LS) and high salinity (HS) stresses in *C. savignyi*. (**C**) Transcript expression level of two isoforms of alternatively spliced gene (*Cs_SRSF12*) after 24 h of control and HT stress groups. (**D**) Percentage of two isoforms of *Cs_SRSF12* gene. (**E**) Conserved domains of isoform1 (upper) and isoform2 (lower).

In the case of *C. robusta*, we excluded *Cr_SRSR7a* and *Cr_SRSR6b* from subsequent gene expression analysis due to their low expression levels. Following recurrent high salinity (HS) stresses, we observed a general increase in the amplitude of gene expression response under the second stress when compared to the first. Furthermore, the overall downregulated expression of SRSF genes at 6 and 24 h shifted towards an upregulated status at the recovery stage of the second stress (Figure 4A). We identified two significantly differentially expressed genes (DEGs): *Cr_SRSF1b* displayed a significant upregulation at HS_1_6, whereas *Cr_SRSF6c* exhibited a significant downregulation at HS_1_6 and HS_2_6.

In *C. savignyi*, we observed that the extent of the gene expression response in SRSF genes induced by temperature stress was generally greater than that induced by salinity stress (Figure 4B). After cold stresses (LT), *Cs_SRSF2* exhibited significant activation at 48 h, whereas *Cs_SRSF3* showed significant repression at 1 h. Following high-temperature stresses (HT), *Cs_SRSF7a* and *Cs_SRSF6b* were significantly downregulated at multiple timepoints, whereas *Cs_SRSF12* displayed an upregulated expression profile. Under low salinity (LS) stress conditions, the expression of *Cs_SRSF1a* and *Cs_SRSF3* significantly decreased at 24 and 48 h, respectively, and Cs_SRSF6a consistently exhibited decreased expression at 24 and 48 h. After high salinity (HS) stress, only *Cs_SRSF2* showed a significant induction at 48 h.

In our analysis of AS profiles of SRSF genes, we found no evidence of DASE across all SRSF genes under various environmental stresses in both species, as determined by the rMATS software. However, at the isoform level, we observed a DASG, *Cs_SRSF12*, following 24 h of HT stress, using the 3D RNA-seq APP (Figure 4C). The *Cs_SRSF12* gene comprised two transcript isoforms, with isoform 1 possessing a complete RRM domain. Notably, the expression of isoform 1 significantly increased at HT24, making it the dominant proportion when compared with isoform 2, which lacks an RRM domain (Figure 4C–E).

## 3. Discussion

Recently, the regulatory roles of AS have been gradually elucidated. AS regulations have been functionally demonstrated to play pivotal roles both in adaptive evolution over extended evolutionary timescales and in responding to specific environmental challenges within a single generation or even shorter time frames [2,22,28]. In this study, we employed the closely related invasive ascidians, *C. robusta* and *C. savignyi*, as a model system to investigate AS response patterns in these two species under various environmental stresses and to explore potential factors contributing to the observed AS variation. Our results unveiled notable variations in AS patterns between the two species when exposed to the same salinity stress, indicating species-specific AS patterns (Figure 2A and Table S1). Moreover, we observed AS variations in response to different environmental challenges (salinity and temperature) within the same species, indicating stress-specific AS patterns (Figure 2A and Table S1). These interspecific AS differences were associated with divergence in genomic architectural features between the two species, such as the intron–exon structure and intron–exon size. Through genome-wide identification of crucial trans-splicing factors (SRSFs), we noted that *C. robusta* possessed two additional SRSF6 members in comparison to *C. savignyi* (Table 2). As expected, all SRSF members exhibited conservation in gene structure and functional domains between *C. robusta* and *C. savignyi*. While we did not detect significantly alternatively spliced SRSFs, our analysis revealed the involvement of other splicing factors and spliceosome component genes that were under AS regulation in response to environmental challenges.

### 3.1. Species-Specific and Environmental Context-Dependent AS Patterns

AS is widespread across eukaryotes, whereas the dominant AS event types and prevalence vary substantially among species [29]. For instance, IR is a major AS event type in plants, while ES is the most common AS type in animals [30]. We identified distinct species-specific AS patterns even when subjected to the same environmental challenges. Our findings revealed that in response to recurrent high salinity stresses, 21.5% of genes exhibited alternative splicing in *C. robusta*, a lower proportion compared to *C. savignyi*, which showed an ASP of 37.7%. Additionally, the ES type accounted for 92.2% of *C. robusta* and 70.7% of *C. savignyi*, aligning with similar findings in other studies involving these species (e.g., [31]). These species-specific AS responses parallel the observations in two related mustard species, *Arabidopsis thaliana* and *Boechera depauperata*, which exhibited differential AS patterns during heat stresses [32]. Furthermore, we conducted a comparative analysis of AS profiles under temperature and salinity stresses in *C. savignyi*, revealing distinct AS responses characterized by higher ASP, ASL, and a greater number of affected genes under temperature stress compared to salinity stress. This aligns with the established notion that heat stresses typically induce more robust AS regulation responses than other forms of stresses [33]. A similar stress-dependent AS response was also documented in the model plant *A. thaliana*, which exhibited varying AS patterns in response to different external and internal stimuli [34]. An intriguing discovery from our study was the dynamic nature of AS response genes, which varied with the duration of stresses and different stress cycles. This dynamic process adds an additional layer of complexity to AS regulation, highlighting the intricate mechanisms governing AS in response to environmental challenges. There are debates regarding the biological consequences of these stress-induced AS variations. Some studies have considered them as nonfunctional transcriptional noises [35], while many have proposed that they might represent useful standing variations that could be subsequently acted upon by natural selection to generate adaptive phenotypes, akin to genetic mutations [1].

### 3.2. Genome Architecture Potentially Underlies AS Variation

Studies have demonstrated that in vertebrates, most species-specific AS patterns were primarily directed by *cis*-regulatory elements [20]. Notably, the frequency of exon skipping has been found to co-evolve with genome architecture [36]. In the case of *C. robusta* and *C. savignyi*, a comparison of their genomes has unveiled extensive variations [37,38]. These divergent genome architecture features likely underlie the AS variations observed in these two species. Indeed, our analysis identified disparities in exon–intron structures and exon–intron size distributions between these two species (Figure 2). Generally, an increased number of introns allows for the generation of a greater number of transcript isoforms [39]. A previous study of ours demonstrated that genes undergoing alternative splicing tended to have more exons [16]. Therefore, the higher proportion of genes with more than ten exons in the *C. savignyi* genome may contribute to its elevated ASP when compared to *C. robusta*. Furthermore, both the length of exons and introns are pivotal parameters that influence AS profiles [36]. Shorter exons were associated with higher exon skipping frequencies across eukaryotes, and exons that exhibit exon skipping tended to be flanked by longer introns [36]. Consequently, the exon skipping frequency correlated with a higher intron-to-exon length ratio [36]. Our results revealed that *C. savignyi* had shorter exons and longer introns, a pattern that could elucidate its heightened ASP and ASL in comparison to *C. robusta.* Additionally, the mean intron size was known to exhibit a positive correlation with ASP and ASL in a genome [27]. However, we did not observe significant correlations between ASL and the mean intron length of DASGs when calculating the Pearson Correlation Coefficient.

The additional peak in intron length distribution is evolutionarily conserved among fungi, vertebrates, and higher plant species [40]. However, the relative size of the major and minor peaks varies across different lineages. For instance, most fungi and plant species typically have smaller introns, whereas vertebrates tend to have a greater number of large introns. The bimodal distribution of introns observed in both *C. robusta* and *C. savignyi* resembled that of teleosts [41], albeit with shorter small introns (58 bp for *C. robusta* and 59 bp for *C. savignyi*, compared to 76 bp for teleosts). In contrast, the additional minor peak in the exon length distribution was not as commonly observed as that in introns, although a similar distribution mode has been reported in the human genome, with the minor peak occurring at similar positions [42]. These differences in intron and exon sizes may involve distinct spliceosome machineries.

### 3.3. Trans-Acting Factors under AS Control in Response to Environmental Stresses

SRSFs and hnRNPs represent critical splicing regulators that play pivotal roles in recruiting the splicing machinery to splice sites. This is especially true for SRSFs, whose involvement in AS regulation during stress responses has been experimentally validated [43,44]. Comparative analysis of SRSFs across 27 eukaryotic species has suggested that the SRSF gene family exhibited evolutionary conservation in terms of sequence and functional domain organization but displayed variability in terms of gene number [45]. Typically, an increase in the number of SRSFs correlated with a more complex AS profile [21]. Our analysis revealed the presence of three additional SRSFs in the whole genome of *C. robusta*, bringing the total count to 11 SRSFs, compared to 8 in a previous study [39]. Nine SRSFs were identified in *C. savignyi*, whereas an additional serine/arginine-related protein 53 (SRRP53) was identified as a crucial *trans*-acting factor under AS control in the response to high- and low-temperature stresses (Table 1). A protein domain analysis indicated that SRRP53 possessed only one RS domain, which differentiates it from canonical SRSFs due to its lack of the RNA recognition motif (RRM) domain. Consequently, SRRP53 falls under the category of SR-related proteins, which also play significant roles in the splicing process [46]. Regarding the four expanded SRSF6 genes in *C. robusta*, we conducted a multiple sequence alignment using their amino acid sequences to elucidate the evolutionary expansion mechanisms of the SRSF4/5/6 subfamily. Despite sharing similar protein domain compositions, the pairwise sequence identity among these genes ranged from 46.24% to 69.29%, falling below the widely accepted threshold for defining duplicated genes (>80%). This suggests that these genes might represent ancient duplication and divergence events. A phylogenetic analysis tracing the evolutionary history of the SRSF4/5/6 subfamily across animals and fungi revealed that the single ancestor SRSF5 gene was not duplicated until the chordate lineage (sea squirt), followed by a subsequent duplication in vertebrates [47]. Therefore, the expansion of the SRSF4/5/6 subfamily in *C. robusta* represents an important evolutionary node for the diversification of SRSF genes and their functions related to AS.

The gene expression of SRSFs has been widely validated to be regulated by environmental stresses [48,49]. Our results also demonstrated significant changes in the expression of several SRSFs. However, it was challenging to establish a direct link between this expression pattern and the genome-wide AS change profiles. One crucial aspect of AS regulation to consider is that SRSFs can be targets of AS regulation themselves, or they can influence the AS of other SRSF members. This phenomenon is widely reported as a prevalent feature of AS regulation [50,51]. Surprisingly, in this study, we did not detect any DASEs in SRSF genes in both *C. robusta* and *C. savignyi* under any environmental conditions. Only one transcript change event was identified in *Cs_SRSF12* at HT24 using the 3DRNAseq v2.0.1 tool. These findings suggest that AS regulations of SRSF genes in the stress response of congeneric *Ciona* species may not be as common as observed in other organisms. In addition to the important roles in constitutive and alternative splicing, SRSFs are also involved in various mRNA metabolism processes, such as mRNA export and stability, nonsense-mediated mRNA decay (NMD), translation, and many others. [52]. Instead of SRSFs, we identified a set of other splicing factors and spliceosome component genes under AS control, including members of hnRNPs. This gene list provides potential candidates for further research into *trans*-acting factors beyond SRSFs. These findings are expected to complement our understanding of rapid responses to environmental challenges, offering insights beyond the mechanisms centered on SRSFs.

### 3.4. Future Perspective

Investigations into causes for AS patterns open a window to better understand how AS plays functional roles in linking genotypes with phenotypes. In addition to genome architecture and splicing factors such as SRSFs and hnRNPs investigated in this study, AS is simultaneously controlled by the core spliceosome, splice site, RNA secondary code, RNA polymerase speed, and other epigenetic regulations [53]. The intricate involvement of such a multitude of factors, along with their potential interactions, presents a significant technical challenge in understanding the causes and consequences of inter- and intra-specific AS variation. Unlike gene expression studies, where changes in expression levels can be easily quantified using short-read sequencing and bioinformatic tools, AS mechanism studies have lagged behind due to these challenges. The recent advancements in long-read sequencing techniques and the availability of high-quality genome assemblies have opened up new possibilities for AS mechanism studies. Full-length transcriptome sequencing using long-read technology can overcome the limitations of short-read sequencing, providing unprecedented opportunities to explore the intricacies of AS regulation in response to environmental stresses and environmental adaptation. The study of AS mechanisms in response to environmental stresses is a complex but evolving field. As technology continues to advance, researchers are better equipped to unravel the intricate web of factors that govern AS and its functional roles in linking genotypes to phenotypes in changing environmental conditions.

## 4. Materials and Methods

### 4.1. Sample Collection

To study the underlying AS regulatory mechanisms under recurrent environmental changes during the ascidian invasion process, we designed an experiment simulating recurrent high salinity stresses using *C. robusta*, which was similar to our another previously published study [17], with low-temperature stresses replaced with high salinity challenges (see detailed experimental design in [17]). Briefly, *C. robusta* individuals were first acclimated to a laboratory environment with a salinity of 30‰ for a week and then subjected to two rounds of high salinity stress (40‰, HS)-recovery to unstressed condition (R). Each phase lasted for 24 h, and three ascidian individuals were collected at seven timepoints, including the end of acclimatization (Control, C), 6 and 24 h at the first HS stress (HS_1_6, HS_1_24), the end of the first recovery (R1), 6 and 24 h at the second HS stress (HS_2_6, HS_2_24), and the end of the second recovery (R2).

### 4.2. RNA Extraction and Sequencing

After sample preparation, the total RNA was isolated using a Trizol reagent (Ambion, Austin, TX, USA) following the manufacturer’s protocol. RNA quality was assessed using Agilent 2100 Bioanalyzer (Agilent Technologies, Santa Clara, CA, USA). A total of 21 strand-specific cDNA libraries were constructed according to the SMART library construction method and sequenced on an Illumina X Ten sequencing platform (Illumina, San Diego, CA, USA) using the 150 bp pair-end reads. The adaptor and low-quality sequences were first filtered out from raw reads using Trimmomatic version 0.36, and the clean reads were mapped to the reference genome (Ensembl KH.109) using Hisat v2.2.1 [54]. The mapped reads were subsequently assembled into the transcripts and the abundance of gene expression was estimated using StringTie v2.1.4 software with default settings [55]. Differential expression analysis was performed between each treatment group and control group using the R v3.6 package DESeq2 v1.40.2 [56], resulting in six pairwise comparisons including HS_1_6 vs. C, HS_1_24 vs. C, R1 vs. C, HS_2_6 vs. C, HS_2_24 vs. C, R2 vs. C, and only the treatment group label was retained for simplicity when it comes to differential analysis. As defined by widely accepted cutoff values, differentially expressed genes (DEGs) were those with a fold change value larger than two as well as a false discovery rate (FDR) lower than 0.05.

### 4.3. AS Profile Changes of C. robusta to Recurrent High Salinity Stresses

Similarly, we used rMATS v.4.0.2 software to detect alternatively sliced events (ASEs) of five different AS types [57], and the proportion of each AS type was calculated by the ASE number of particular AS type/all ASE number detected in a pairwise comparison of rMATS analysis. Differentially alternatively spliced events (DASEs) in response to recurrent HS stresses were defined using |ΔPSI (the difference in inclusion level of certain alternative splicing sites between treatment and control groups)| > 10% and adjusted *p* value < 0.05. If at least one DASE occurred on one gene, it would be defined as differentially alternatively spliced gene (DASG). After the list of DASGs was obtained, gene ontology (GO) functional enrichment analysis of DASGs was conducted using a hypergeometric test using online Omicshare CloudTools (http://www.omicshare.com/tools, assessed on 2 March 2023).

Genome-wide alternative splicing prevalence (ASP) and level (ASL) were calculated separately under different environments. ASP is the proportion of alternatively spliced genes in the whole genome, whereas ASL is the average number of ASEs per alternatively spliced gene. 

### 4.4. Transcriptomic Data Collection of C. savignyi

Genome-wide gene expression and alternative splicing analysis were retrieved from our previously published results [16,17]. Briefly, *C. savignyi* individuals were separately challenged using four types of environmental challenges, including low temperature (LT), high temperature (HT), low salinity (LS), and high salinity (HS). Individuals were sampled at 1, 24, and 48 h during each treatment, which were labeled as “treatment-timepoint” such as LT1, LT24, and LT48. All bioinformatic analyses were the same as those in *C. robusta*.

### 4.5. Genomic Distribution of the Number of Exons and Length of Exons and Introns

To test potential genomic characteristics underlying different alternative splicing profiles in *C. robusta* and *C. savignyi*, the number of exons per gene and length of exons and introns across the whole genome were extracted from the genome annotation files in Ensembl using the R v4.3 package GenomicFeatures v1.52.2, and then illustrated by ggplot2 v2.1.0 package. GO enrichment analysis of the genes with a single exon and more than ten exons was conducted using online Omicshare CloudTool (http://www.omicshare.com/tools, assessed on 2 March 2023)., and after obtaining the list of significant GO terms of biological process category, redundant GO terms were removed by REVIGO website (http://revigo.irb.hr/, assessed on 2 March 2023).

### 4.6. Identification of SRSF Gene Family Members

The reference proteomes of *C. robusta* and *C. savignyi* were downloaded from the Ensembl website and then submitted to EggNOG v6.0 (http://eggnog6.embl.de/, assessed on 21 February 2023) for comprehensive functional annotation. First, we searched for SRSF genes from the annotation results and used each SRSF member in *C. robusta* (or *C. savignyi*) as a query to do BLAST against the proteomes of *C. savignyi* (or *C. robusta*) for all potential orthologs. Secondly, the Hidden Markov Model (HMM) profiles of RRM (PF00076 and PF13893) and Arginine/serine-rich protein (PF17069 and PF15996) were downloaded from Pfam database, and we searched these four domains against the proteome database of *C. robusta* and *C. savignyi* using HMMER 3.0 with an E-value < 1 × 10^−6^, and only the proteins with both domains were retained for subsequent analyses. The identified SRSF genes were renamed using three parts of information, species (Cr: *Ciona robusta*; Cs: *Ciona savignyi*) + SRSF member + one additional lowercase letter if this SRSF member has more than one copy. All obtained candidate SRSF members were further verified for the presence of both RRM and RS domains by the SMART database. The density of RS repeats in the RS domain was tested by the LCD-Composer web server [46].

The longest peptide isoform of all identified SRSF members of *C. robusta* and *C. savignyi* were aligned by MUSCLE, and then the maximum likelihood (ML) phylogenetic tree was constructed using MEGA X to illustrate the homologous relationship among orthologs or paralogs. The exon–intron structures of all SRSF genes were illustrated using Gene Structure Display Server 2.0 by aligning the cDNA sequences with the corresponding genomic DNA sequences [58].

### 4.7. Gene Expression and Alternative Splicing Responses to Environmental Changes

Gene expression and alternative splicing changes of all SRSF genes in response to environmental stresses were extracted from the above RNA-seq analysis. In addition to AS analyses by rMATS, we also used a 3D RNA-seq v2.0.1 tool to study AS at the transcript isoform level [59].

## 5. Conclusions

Using congeneric invasive *C. robusta* and *C. savignyi* as a model, we assessed and further compared the alternative splicing prevalence, type, and response patterns to environmental challenges (salinity and temperature stresses) during biological invasions. Our results showed species-specific and environmental context-dependent patterns in closely related ascidians. Divergent genome architecture might underlie the observed AS variation between these two species. Surprisingly, the traditionally believed important splicing factors in SRSFs did not show prevalent AS regulations during environmental stress response, but we identified a list of other splicing factors and spliceosome components under AS control as potential functional *trans*-acting factors. Such a gene list provides potential candidates to further study AS-mediated rapid responses to environmental challenges complementary to SRSFs.

## Figures and Tables

**Table 1:** The list of differentially alternative spliced genes (DASGs) functionally associated with alternative splicing regulation in *Ciona robusta* and *C. savignyi*.

Species	Stress	Gene ID ^a^	Gene Name ^b^	Function	Stress-Induced AS Change	Exon Size (bp)
*Ciona robusta*	High salinity	ENSCING00000001244	*hnRNPLL*	Recognition of splicing silencer	Skipping the 7th exon at HS_1_6 and HS_2_6	74
		ENSCING00000002697	*hnRNPK*	Recognition of splicing silencer	Inclusion of the 4th exon at HS_1_24 and R1	44
		ENSCING00000007926	*CLK2*	Phosphorylation of SRSF proteins	Inclusion of the 4th exon at HS_1_6	103
*Ciona savignyi*	High salinity	None				
	Low salinity	ENSCSAVG00000000753	*hnRNPM*	Recognition of splicing silencer	Inclusion of the 4th exon at LS48	8
		ENSCSAVG00000007687	*SNRPA1*	Component of the spliceosome	Skipping the 4th exon at LS1 and LS48	80
		ENSCSAVG00000009055	*BBP*	Required for pre-spliceosome formation	Skipping the 3rd exon at LS24	76
	High temperature	ENSCSAVG00000003682	*SRRP53*	Recognition of the 3’ splice site	Skipping the 10th exon at HT1	174
		ENSCSAVG00000000753	*hnRNPM*	Pre-mRNA binding protein	Skipping the 5th exon at HT1	94
		ENSCSAVG00000004927	*SLU7*	Component of the spliceosome	Skipping the 6th exon at HT1	127
		ENSCSAVG00000007421	*PTBP2*	Negative regulation of exons splicing	Skipping the 8th exon at HT1	34
		ENSCSAVG00000007539	*PHF5A*	Component of the minor spliceosome	Skipping the 2nd exon at HT1	24
		ENSCSAVG00000009443	*PRPF38B*	Required for pre-mRNA splicing	Skipping the 2nd exon at HT1	266
	Low temperature	ENSCSAVG00000001385	*PUF60*	Binds to the pyrimidine tract and 3’-splice site regions of pre-mRNA	Skipping the 4th exon at LT24	84
		ENSCSAVG00000003682	*SRRP53*	Recognition of the 3’ splice site	Skipping the 10th exon at LT48	174

a: all gene ID from Ensembl database; b: the abbreviations of gene name, *hnRNPLL*: Heterogeneous nuclear ribonucleoprotein L; *hnRNPK*: Heterogeneous nuclear ribonucleoprotein K; *CLK2*: Dual specificity protein kinase; *hnRNPM*: heterogeneous nuclear ribonucleoprotein M; *SNRPA1*: U2 small nuclear ribonucleoprotein A’-like; *BBP*: Branchpoint-bridging protein; *SRRP53*: serine/arginine-related protein 53; *SLU7*: SLU7 Pre-mRNA-splicing factor; *PTBP2*: Polypyrimidine tract-binding protein 2; *PHF5A*: PHD finger-like domain-containing protein 5A; *PRPF38B*: Pre-mRNA-splicing factor 38; *PUF60*: Poly(U)-binding-splicing factor PUF60.

**Table 2:** Summary information of serine/arginine-rich splicing factors (SRSFs) in *Ciona robusta* and *C. savignyi*.

Species	Family	Gene ID	Gene Name	Genomic Location	Protein	RRM Postion	SR Density %
*Ciona savignyi*	SRSF1/9	ENSCSAVG00000003567	*Cs_SRSF1a*	reftig_15: 65,169–68,621	165	58–129	61.22
		ENSCSAVG00000006093	*Cs_SRSF1b*	reftig_65: 806,958–812,464	233	10–79, 111–177	59.38
	SRSF2/8	ENSCSAVG00000009199	*Cs_SRSF2*	reftig_0: 1,818,675–1,820,985	178	16–89	64.29
	SRSF3/7	ENSCSAVG00000002676	*Cs_SRSF3*	reftig_11: 177,090–179,396	183	12–80	58.77
		ENSCSAVG00000007369	*Cs_SRSF7a*	reftig_26: 994,499–997,745	246	13–81	53.37
		ENSCSAVG00000010868	*Cs_SRSF7b*	reftig_19: 4,084,425–4,086,767	220	6–74	64.08
	SRSF4/5/6	ENSCSAVG00000002581	*Cs_SRSF6a*	reftig_113: 112,440–114,648	289	5–70, 130–198	69.23
		ENSCSAVG00000011376	*Cs_SRSF6b*	reftig_48: 3,543,330–3,544,136	252	8–73, 129–197	75.44
	SRSF10/12	ENSCSAVG00000005130	*Cs_SRSF12*	reftig_134: 409,162–425,269	226	9–82	48.55
*Ciona robusta*	SRSF1/9	ENSCING00000000257	*Cr_SRSF1a*	Chromosome 8: 137,156–138,366	167	53–124	60.71
		ENSCING00000007375	*Cr_SRSF1b*	Chromosome 8: 4,297,332–4,299,772	235	10–79, 111–182	57.58
	SRSF2/8	ENSCING00000004198	*Cr_SRSF2*	Scaffold HT000041.1: 85,198–86,831	166	16–89	60.81
	SRSF3/7	ENSCING00000011663	*Cr_SRSF3*	Chromosome 3: 1,159,348–1,160,853	149	12–80	55.41
		ENSCING00000003530	*Cr_SRSF7a*	Chromosome 14: 2,961,797–2,963,511	251	16–84	61.9
		ENSCING00000023459	*Cr_SRSF7b*	Chromosome 2: 418,131–420,268	230	6–74	64.65
	SRSF4/5/6	ENSCING00000012370	*Cr_SRSF6a*	Scaffold HT000150.1: 282,453–285,903	292	5–70, 130–180	68.37
		ENSCING00000020631	*Cr_SRSF6b*	Chromosome 5: 3,372,757–3,373,533	243	5–70, 124–192	69.81
		ENSCING00000024718	*Cr_SRSF6c*	Chromosome 5: 3,383,599–3,385,855	342	5–70, 125–193	64.47
		ENSCING00000011362	*Cr_SRSF6d*	Chromosome 2: 6,628,454–6,629,329	283	5–75, 118–191	51.49
	SRSF10/12	ENSCING00000018401	*Cr_SRSF12*	Chromosome 8: 1,554,382–1,558,494	227	9–82	49.19

## Data Availability

All raw sequencing data was deposited in the National Center for Biotechnology Information (NCBI) Sequence Read Archive (SRA) database under the accession number SRP152910 and SRP460043.

## Supplementary Materials

The following supporting information can be downloaded at: https://www.mdpi.com/article/10.3390/ijms241914921/s1.

## References

[1] Wright C.J., Smith C.W.J., Jiggins C.D. (2022). Alternative splicing as a source of phenotypic diversity. Nat. Rev. Genet..

[2] Singh P., Ahi E.P. (2022). The importance of alternative splicing in adaptive evolution. Mol. Ecol..

[3] Jacobs A., Elmer K.R. (2021). Alternative splicing and gene expression play contrasting roles in the parallel phenotypic evolution of a salmonid fish. Mol. Ecol..

[4] Verta J.P., Jacobs A. (2022). The role of alternative splicing in adaptation and evolution. Trends Ecol. Evol..

[5] Merkin J., Russell C., Chen P., Burge C.B. (2012). Evolutionary dynamics of gene and isoform regulation in mammalian tissues. Science.

[6] Zhao F., Yan Y., Wang Y., Liu Y., Yang R. (2023). Splicing complexity as a pivotal feature of alternative exons in mammalian species. BMC Genomics.

[7] Griggio F., Voskoboynik A., Iannelli F., Justy F., Tilak M.K., Turon X., Pesole G., Douzery E.J., Mastrototaro F., Gissi C. (2014). Ascidian mitogenomics: Comparison of evolutionary rates in closely related taxa provides evidence of ongoing speciation events. Genome Biol. Evol..

[8] Zhan A., Briski E., Bock D.G., Ghabooli S., MacIsaac H.J. (2015). Ascidians as models for studying invasion success. Mar. Biol..

[9] Tarallo A., Yagi M., Oikawa S., Agnisola C., D’Onofrio G. (2016). Comparative morpho-physiological analysis between *Ciona robusta* and *Ciona savignyi*. J. Exp. Mar. Biol. Ecol..

[10] Chen Y., Shenkar N., Ni P., Lin Y., Li S., Zhan A. (2018). Rapid microevolution during recent range expansion to harsh environments. BMC Evol. Biol..

[11] Zhang Z., Capinha C., Karger D.N., Turon X., MacIsaac H.J., Zhan A. (2020). Impacts of climate change on geographical distributions of invasive ascidians. Mar. Environ. Res..

[12] Chen Y., Ni P., Fu R., Murphy K.J., Wyeth R.C., Bishop C.D., Huang X., Li S., Zhan A. (2022). (Epi)genomic adaptation driven by fine geographical scale environmental heterogeneity after recent biological invasions. Ecol. Appl..

[13] Huang X., Li S., Ni P., Gao Y., Jiang B., Zhou Z., Zhan A. (2017). Rapid response to changing environments during biological invasions: DNA methylation perspectives. Mol. Ecol..

[14] Ni P., Murphy K.J., Wyeth R.C., Bishop C.D., Li S., Zhan A. (2019). Significant population methylation divergence and local environmental influence in an invasive ascidian *Ciona intestinalis* at fine geographical scales. Mar. Biol..

[15] Huang X., Zhan A. (2021). Highly dynamic transcriptional reprogramming and shorter isoform shifts under acute stresses during biological invasions. RNA Biol..

[16] Huang X., Li H., Zhan A. (2023). Multidimensional plasticity jointly contributes to rapid acclimation to environmental challenges during biological invasions. RNA.

[17] Li H., Huang X., Zhan A. (2020). Stress Memory of Recurrent Environmental Challenges in Marine Invasive Species: *Ciona robusta* as a Case Study. Front. Physiol..

[18] Li X., Li S., Cheng J., Fu R., Zhan A. (2021). Proteomic Response to Environmental Stresses in the Stolon of a Highly Invasive Fouling Ascidian. Front. Mar. Sci..

[19] Black D.L. (2003). Mechanisms of alternative pre-messenger RNA splicing. Annu. Rev. Biochem..

[20] Barbosa-Morais N.L., Irimia M., Pan Q., Xiong H.Y., Gueroussov S., Lee L.J., Slobodeniuc V., Kutter C., Watt S., Colak R. (2012). The evolutionary landscape of alternative splicing in vertebrate species. Science.

[21] Busch A., Hertel K.J. (2012). Evolution of SR protein and hnRNP splicing regulatory factors. Wiley Interdiscip Rev. RNA.

[22] Laloum T., Martín G., Duque P. (2018). Alternative Splicing Control of Abiotic Stress Responses. Trends Plant Sci..

[23] Long J.C., Caceres J.F. (2009). The SR protein family of splicing factors: Master regulators of gene expression. Biochem. J..

[24] Manley J.L., Krainer A.R. (2010). A rational nomenclature for serine/arginine-rich protein splicing factors (SR proteins). Genes Dev..

[25] de Oliveira Freitas Machado C., Schafranek M., Bruggemann M., Hernandez Canas M.C., Keller M., Di Liddo A., Brezski A., Blumel N., Arnold B., Bremm A. (2023). Poison cassette exon splicing of SRSF6 regulates nuclear speckle dispersal and the response to hypoxia. Nucleic Acids Res..

[26] Kataoka N., Matsumoto E., Masaki S. (2021). Mechanistic Insights of Aberrant Splicing with Splicing Factor Mutations Found in Myelodysplastic Syndromes. Int. J. Mol. Sci..

[27] Yang P., Wang D., Kang L. (2021). Alternative splicing level related to intron size and organism complexity. BMC Genom..

[28] Ling Y., Mahfouz M.M., Zhou S. (2021). Pre-mRNA alternative splicing as a modulator for heat stress response in plants. Trends Plant Sci..

[29] Schwartz S.H., Silva J., Burstein D., Pupko T., Eyras E., Ast G. (2008). Large-scale comparative analysis of splicing signals and their corresponding splicing factors in eukaryotes. Genome Res.

[30] Kalyna M., Simpson C.G., Syed N.H., Lewandowska D., Marquez Y., Kusenda B., Marshall J., Fuller J., Cardle L., McNicol J. (2012). Alternative splicing and nonsense-mediated decay modulate expression of important regulatory genes in Arabidopsis. Nucleic Acids Res.

[31] Chen L., Bush S.J., Tovar-Corona J.M., Castillo-Morales A., Urrutia A.O. (2014). Correcting for differential transcript coverage reveals a strong relationship between alternative splicing and organism complexity. Mol. Biol. Evol..

[32] Kannan S., Halter G., Renner T., Waters E.R. (2018). Patterns of alternative splicing vary between species during heat stress. AoB Plants.

[33] Bond U. (1988). Heat shock but not other stress inducers leads to the disruption of a sub-set of snRNPs and inhibition of in vitro splicing in HeLa cells. EMBO J..

[34] Martin G., Marquez Y., Mantica F., Duque P., Irimia M. (2021). Alternative splicing landscapes in Arabidopsis thaliana across tissues and stress conditions highlight major functional differences with animals. Genome Biol..

[35] Tress M.L., Abascal F., Valencia A. (2017). Alternative Splicing May Not Be the Key to Proteome Complexity. Trends Biochem Sci.

[36] Grau-Bove X., Ruiz-Trillo I., Irimia M. (2018). Origin of exon skipping-rich transcriptomes in animals driven by evolution of gene architecture. Genome Biol..

[37] Delsuc F., Philippe H., Tsagkogeorga G., Simion P., Tilak M.K., Turon X., Lopez-Legentil S., Piette J., Lemaire P., Douzery E.J.P. (2018). A phylogenomic framework and timescale for comparative studies of tunicates. BMC Biol..

[38] Satou Y., Nakamura R., Yu D., Yoshida R., Hamada M., Fujie M., Hisata K., Takeda H., Satoh N. (2019). A Nearly Complete Genome of *Ciona intestinalis* Type A (*C. robusta*) Reveals the Contribution of Inversion to Chromosomal Evolution in the Genus *Ciona*. Genome Biol. Evol..

[39] Kim N., Alekseyenko A.V., Roy M., Lee C. (2007). The ASAP II database: Analysis and comparative genomics of alternative splicing in 15 animal species. Nucleic Acids Res..

[40] Wu J., Xiao J., Wang L., Zhong J., Yin H., Wu S., Zhang Z., Yu J. (2013). Systematic analysis of intron size and abundance parameters in diverse lineages. Sci. China Life Sci..

[41] Jakt L.M., Dubin A., Johansen S.D. (2022). Intron size minimisation in teleosts. BMC Genom..

[42] Piovesan A., Caracausi M., Antonaros F., Pelleri M.C., Vitale L. (2016). GeneBase 1.1: A tool to summarize data from NCBI gene datasets and its application to an update of human gene statistics. Database.

[43] Zhao X., Tan L., Wang S., Shen Y., Guo L., Ye X., Liu S., Feng Y., Wu W. (2021). The SR Splicing Factors: Providing Perspectives on Their Evolution, Expression, Alternative Splicing, and Function in *Populus trichocarpa*. Int. J. Mol. Sci..

[44] Zhang W., Du B., Liu D., Qi X. (2014). Splicing factor SR34b mutation reduces cadmium tolerance in Arabidopsis by regulating iron-regulated transporter 1 gene. Biochem. Biophys. Res. Commun..

[45] Richardson D.N., Rogers M.F., Labadorf A., Ben-Hur A., Guo H., Paterson A.H., Reddy A.S.N. (2011). Comparative Analysis of Serine/Arginine-Rich Proteins across 27 Eukaryotes: Insights into Sub-Family Classification and Extent of Alternative Splicing. PLoS ONE.

[46] Cascarina S.M., Ross E.D. (2022). Expansion and functional analysis of the SR-related protein family across the domains of life. RNA.

[47] Lareau L.F., Brenner S.E. (2015). Regulation of splicing factors by alternative splicing and NMD is conserved between kingdoms yet evolutionarily flexible. Mol. Biol. Evol..

[48] Wei F., Chen P., Jian H., Sun L., Lv X., Wei H., Wang H., Hu T., Ma L., Fu X. (2022). A Comprehensive Identification and Function Analysis of Serine/Arginine-Rich (SR) Proteins in Cotton (*Gossypium* spp.). Int. J. Mol. Sci..

[49] Yoon E.K., Krishnamurthy P., Kim J.A., Jeong M.-J., Lee S.I. (2018). Genome-wide Characterization of Brassica rapa Genes Encoding Serine/arginine-rich Proteins: Expression and Alternative Splicing Events by Abiotic Stresses. J. Plant Biol..

[50] Gu J., Ma S., Zhang Y., Wang D., Cao S., Wang Z.Y. (2020). Genome-Wide Identification of Cassava Serine/Arginine-Rich Proteins: Insights into Alternative Splicing of Pre-mRNAs and Response to Abiotic Stress. Plant Cell Physiol..

[51] Jin X. (2022). Regulatory Network of Serine/Arginine-Rich (SR) Proteins: The Molecular Mechanism and Physiological Function in Plants. Int. J. Mol. Sci..

[52] Kumar K., Sinha S.K., Maity U., Kirti P.B., Kumar K.R.R. (2022). Insights into established and emerging roles of SR protein family in plants and animals. Wiley Interdiscip. Rev. RNA.

[53] Ding F., Su C.J., Edmonds K.K., Liang G., Elowitz M.B. (2022). Dynamics and functional roles of splicing factor autoregulation. Cell Rep..

[54] Kim D., Langmead B., Salzberg S.L. (2015). HISAT: A fast spliced aligner with low memory requirements. Nat. Methods.

[55] Pertea M., Pertea G.M., Antonescu C.M., Chang T.C., Mendell J.T., Salzberg S.L. (2015). StringTie enables improved reconstruction of a transcriptome from RNA-seq reads. Nat. Biotechnol..

[56] Love M.I., Huber W., Anders S. (2014). Moderated estimation of fold change and dispersion for RNA-seq data with DESeq2. Genome Biol..

[57] Shen S., Park J.W., Lu Z.X., Lin L., Henry M.D., Wu Y.N., Zhou Q., Xing Y. (2014). rMATS: Robust and flexible detection of differential alternative splicing from replicate RNA-Seq data. Proc. Natl. Acad. Sci. USA.

[58] Hu B., Jin J., Guo A., Zhang H., Luo J., Gao G. (2015). GSDS 2.0: An upgraded gene feature visualization server. Bioinformatics.

[59] Guo W., Tzioutziou N., Stephen G., Milne I., Calixto C., Waugh R., Brown J.W.S., Zhang R. (2021). 3D RNA-seq—A powerful and flexible tool for rapid and accurate differential expression and alternative splicing analysis of RNA-seq data for biologists. RNA Biol..

