# Supplementary material for: Interplays between cis- and trans-Acting Factors for Alternative Splicing in Response to Environmental Changes during Biological Invasions of Ascidians
Source: Int J Mol Sci. 2023 Oct 5;24(19):14921. doi: 10.3390/ijms241914921 (PMC10573349; doi:10.3390/ijms241914921)
Supplement: Supplementary file 1 [file ijms-24-14921-s001.zip › ijms-2610187-supplementary.pdf]

Figure S1. GO enrichment analysis of genes with different exon numbers. (A) genes with only one exon in *C. robusta*; (B) genes with only one exon in *C. savignyi*; (C) genes with more than ten exons in *C. robusta*; (D) genes with more than ten exons in *C. savignyi*.

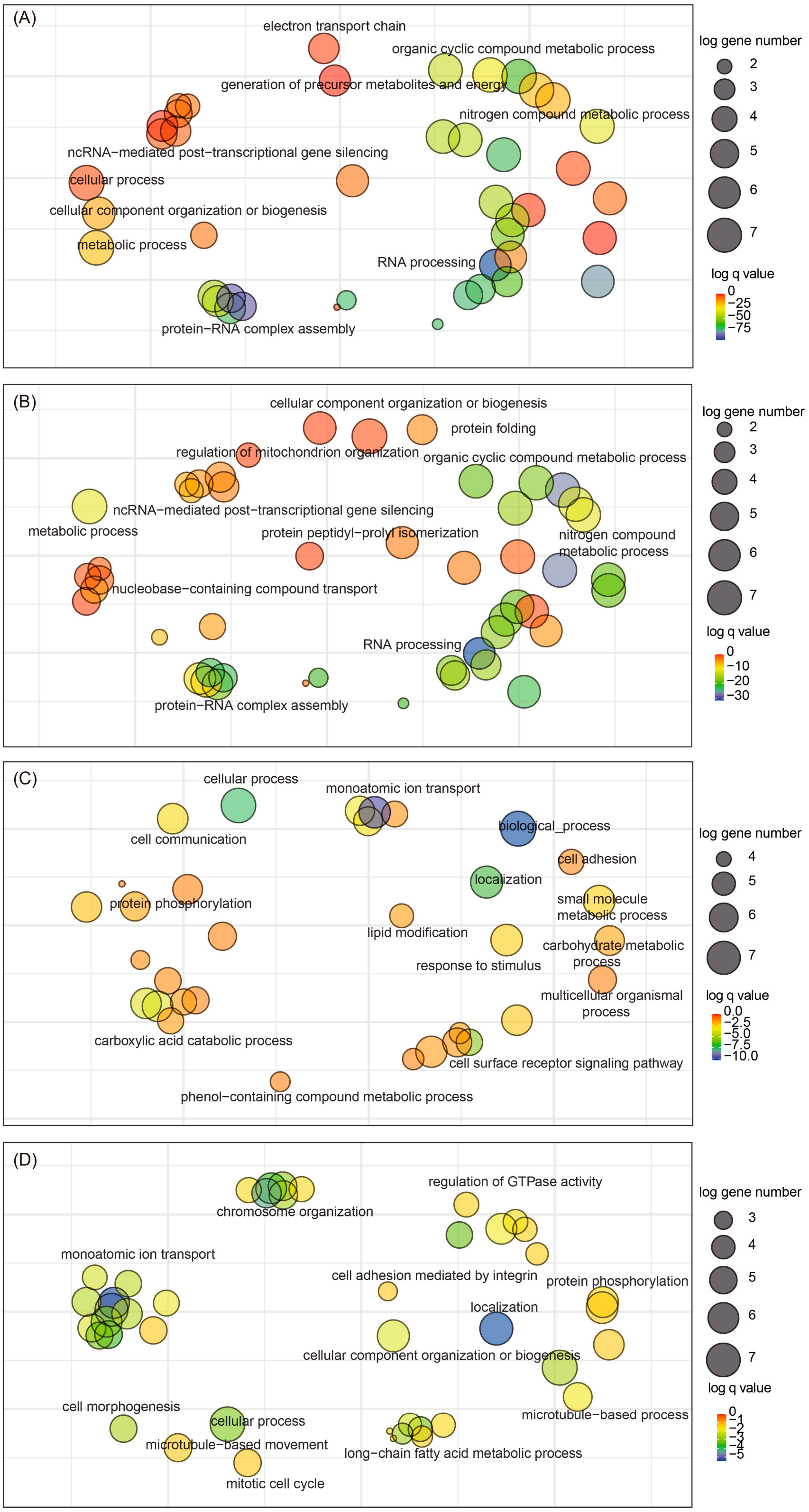

Table S1 Comparisons of different alternative splicing types in congeneric ascidians under different environmental stresses

**Species under stress**	**SE**	**MXE**	**A3SS**	**A5SS**	**RI**
C. robusta under salinity stress	92.2%	5.8%	1.1%	0.8%	0.01%
C. savignyi under salinity stress	70.7%	11.7%	6.96%	10.38%	0.24%
C. savignyi under temperature stress	51.8%	10.2%	11.03%	23.48%	3.41%
